# Patient satisfaction with treatments and outcomes in residential addiction institutions

**DOI:** 10.1177/1455072517718456

**Published:** 2017-08-11

**Authors:** Helle Wessel Andersson, Eli Otterholt, Rolf W. Gråwe

**Affiliations:** St. Olav’s University Hospital, Trondheim, Norway; Møre and Romsdal Hospital Trust, Molde, Norway; St. Olav’s University Hospital, Trondheim, Norway

**Keywords:** patient satisfaction, residential treatment, substance use disorder, treatment outcome, user involvement

## Abstract

**Aim:**

The objective of the present study was to investigate associations between patients’ satisfaction with different domains of inpatient substance use treatment and their perceived treatment outcome. The primary purpose was to identify domains of treatment satisfaction most strongly associated with a positive treatment outcome.

**Design:**

Data were based on a survey among 188 patients with alcohol and/or illicit substance use disorders completing a three–six-month inpatient stay at one of two public clinics in Central Norway. The survey was carried out shortly before discharge. The 15-item questionnaire covered ratings of staff and programme factors, and services received for medical and mental problems and ancillary services. The outcome score was based on items measuring perceived substance use improvements and benefit of treatment.

**Results:**

A significant proportion of patients were dissatisfied with the support provided for housing, financial issues and employment. Confidence in staff competence was the domain of treatment satisfaction most strongly associated with the outcome score. Furthermore, patients were more likely to report a positive outcome when they were actively involved in the treatment, as indicated by satisfaction with opportunities to affect treatment plans.

**Conclusion:**

Our results suggest that patient-experienced improvements are connected to confidence in staff competence and user involvement. The findings may be interpreted as supporting a collaborative relationship between patients and counsellors.

In Norway, multidisciplinary specialised drug treatment focuses comprehensively on patients’ health problems and social situations (Norwegian Directorate of Health, 2009). Although reduction or cessation of drug use is the primary goal of substance use treatment, treatment should aim at improving individuals’ life circumstances in areas that are important for recovery and community adaptation. The majority of patients in residential substance abuse treatment have a substantial number of addiction-related problems such as mental disorders (Bergly, Gråwe, & Hagen, 2014; Kessler, 2004; Landheim, Bakken, & Vaglum, 2006) and social problems that may adversely affect their individual functioning. Comprehensive treatment should provide ancillary services related to domains such as housing, financial issues, and employment, as these are all areas of concern for recovery and reintegration into the community (Duffy & Baldwin, 2013; Laudet & White, 2010; Nordfjærn, Rundmo, & Hole, 2010).

Patients’ autonomy and opportunity to influence the course of their own treatment is crucial in substance abuse treatment (Brener, Resnick, Elard, Treloar, & Bryant, 2009; McCallum, Mikocka-Walus, Gaughwin, Andrews, & Turnbull, 2015; Rance & Treloar, 2015). Establishing a trusting and collaborative relationship between counsellor and patient early on in the treatment course may be a prerequisite for successful implementation of user involvement in substance abuse treatment (Rance & Treloar, 2015). Since 2004, persons with substance use disorders in Norway have legislated rights to specialised treatment equivalent to physical and mental health disorders. A body of patients’ rights laws have also been introduced to ensure the quality of treatment through increased user involvement. Service user involvement builds on the principle that people who use the services are experts on their own treatment needs and on how services can be improved. Involving patients by allowing them to evaluate the services they receive may contribute to the development and delivery of more effective services.

Patient satisfaction with healthcare is an indicator of the quality of service delivery (Trujols, Iraurgi, Oviedo-Joekes, & Guárdia-Olmos, 2014) and important for service programme evaluation and quality (Shipley, Hilborn, Hansel, Tyrer, & Tyrer, 2000; WHO, 2000). It is argued that research on satisfaction in substance abuse treatment should use multiple indicators of treatment satisfaction such as staff and programme factors (Marsden et al., 2000). Based on retrospective data on patients’ satisfaction with different aspects of drug treatment agencies in Scotland, Morris and Gannon (2008) suggest that patients may profit from greater involvement and individualised treatment. Several other studies have suggested that high patient satisfaction is associated with subsequent improved outcomes. This has been shown in methadone maintenance programmes (Deering, Horn, & Frampton, 2012; Kelly, O’Grady, Brown, Mitchell, & Schwarts, 2010; Perreault et al., 2010), outpatient departments (Thylstrup, 2011), 12-step groups (Kendra, Weingardt, Cicciare, & Timko, 2015), and in a mixed sample of patients from community, residential, and prison settings (Morris & McKeganey, 2007).

In most previous studies, satisfaction items are typically averaged and treated as single global satisfaction scores in the analyses, or just based on a single global satisfaction item (Kendra et al., 2015; Morris & McKeganey, 2007; Perreault et al., 2010). Thus, these studies have not provided information about the relative influence of the various aspects of treatment satisfaction on treatment outcome.

Little is currently known about inpatients’ satisfaction with different domains of substance abuse treatment, and few studies have investigated the aspects of patient satisfaction associated with patients’ perceived treatment outcome. The aims of the present study were therefore to assess treatment satisfaction among patients who have completed an inpatient substance abuse treatment programme, with a focus on the association between satisfaction with different aspects of care and self-reported outcome. Our primary purpose was to identify aspects of inpatients’ satisfaction most strongly associated with a positive outcome. Based on previous research (Brener et al., 2009; Morris & Gannon, 2008; Rance & Treloar, 2015), we assumed that patient satisfaction with opportunity to participate in treatment would be associated with a perceived positive treatment outcome.

## Methods

### Study settings

The study was carried out at two public substance use treatment clinics in Central Norway from February 2012 to August 2015. The clinics offer a three–six-month inpatient treatment programme for patients with substance use disorders or substance dependency (alcohol and illicit drugs), referred from social services, general practitioners, or the specialised health services (Ministry of Health Care Services, 2004). The patient population is characterised by polysubstance use, high prevalence of mental health problems, low education, unemployment, and a previous history of inpatient stays (European Monitoring Centre for Drugs and Drug Addiction, 2016; Stallvik, 2015). A comprehensive treatment and recovery programme is directed towards individually based social, biological, and mental health needs, and is provided by a combination of group and individual therapy, including milieu and cognitive behavioural therapies. The programme also prepares the patient for reintegration into the community by providing help with housing, financial issues, and employment.

### Participants and procedure

Measurements of satisfaction were carried out shortly before discharge, when the patients had completed the entire inpatient treatment programme. Among eligible patients a total of 188 (56%) responded to the questionnaire. Reasons for non-completion of the questionnaire were either that it was not administered by the research assistant, or that patients did not want to participate.

### Measures

The measures were based on an instrument developed by the Norwegian Knowledge Centre for the Health Services, commissioned by the Norwegian health authorities (Dahle & Iversen, 2011). The instrument has been used for cross-sectional national reporting of patient satisfaction with inpatient substance abuse treatment (Haugum, Iversen, Bjartnaes, & Lindahl, 2017).

### Outcome measure

The patient-reported outcome score was based on the patients’ responses to the following two items: “Treatment has contributed to stabilising your substance use problems” and “I have benefited from the treatment”. Each item was answered on a five-point Likert scale (0–4), with a higher score indicating more positive outcomes. The correlation between the two items was *r* = 0.59 (*p* < 0.001). Scores on the two items were summed to compute a composite outcome score (range 0–8). The two items contributed equally to the composite outcome variable, as indicated by correlations of *r* = 0.88 and *r* = 0.90, respectively. The outcome score in the sample ranged from 1–8, with a median of 6.0 (*SD* = 1.6).

### Patient satisfaction with staff and programme factors

Ten items targeted satisfaction with different domains of treatment. These items have been found to be especially important for patients with substance use problems (Marsden et al., 2000). The items were answered on a five-point Likert scale (1–5), with a higher score indicating higher satisfaction. The Cronbach’s alpha value of 0.85 indicated good internal consistency reliability. As in other studies, scores on treatment satisfaction were skewed toward positive assessments (i.e., Kendra et al., 2015; Perreault et al., 2010; Zhang, Gerstein, & Friedmann, 2008). The responses were dichotomised into high satisfaction (1) (scores 4–5) and low satisfaction (0) (scores 1–3).

### Satisfaction with specific domains of treatment

Additional items were included to measure patients’ satisfaction in areas that are particularly relevant for patients with substance abuse problems. A total of five items were included to cover experiences with services received for medical and mental health problems, as well as for housing, financial, and employment problems. Patients were asked to rate these aspects of treatment on a three-point scale (1–3), with higher scores indicating higher satisfaction. The responses were dichotomised into high satisfaction (1) (score 3) and low satisfaction (0) (scores 1–2). Because the ancillary services may not be relevant for all patients, it was possible to score “not applicable”.

### Data analysis

Descriptive statistics were used to describe sample characteristics, distribution of satisfaction scores, and perceived treatment outcome. We used Pearson’s bivariate correlation coefficients to reveal the satisfaction items associated with the outcome score. The dichotomised items that had significant bi-variate correlations with the outcome variable were included in a multivariate linear regression analysis. In order to reduce the risk of multicollinearity, only items with a *p*-value less than 0.01 were included in the multivariate model. This model tested the relative role of the different aspect of treatment satisfaction on perceived outcome.

## Results

Sample characteristics are presented in Table 1. The sample comprised more men than women. The majority were 18–29 years old and had completed high school. About half of the sample reported using three or more substances (polysubstance use). Compared with previous Norwegian surveys of patients in residential substance abuse treatment, the current sample appeared to be slightly younger and more were polysubstance users. However, the distribution of gender and educational levels corresponded largely to those of previous studies (Bergly et al., 2014; Haugum & Iversen, 2014).

**Table 1.** Sample demographics.

Demographics	*n*	%
Male	123	65
Female	65	35
18–23 years	60	33
24–29 years	52	29
30–35 years	31	17
> 35 years	37	21
One or two substance types	45	44
Three or more substance types	57	56

*Note*. Age was missing for eight patients. Substance use before index stay was only included in the 2014–2015 cohort.

Table 2 shows the distribution and frequency of low and high satisfaction responses. The two highest levels of high satisfaction reported were on the admission process and perceptions of being treated with courtesy and respect. Next highest were staff available when needed, opportunities to affect treatment plan, and confidence in staff competence. Below these came staff help with motivation, problems understood by staff, and availability of staff counselling. The lowest levels of satisfaction were measured for information provided about ward routines and treatment tailored to individual needs.

**Table 2.** Percentage of low and high responses on patient satisfaction items.

Items	*n*	% Low satisfaction	% High satisfaction
Admission process	187	13	87
Information about ward routines	188	42	58
Treatment tailored to individual needs	185	42	58
Opportunities to affect treatment plan	186	25	75
Treated with courtesy and respect	186	17	83
Availability of staff counselling	188	38	62
Problems understood by staff	187	33	67
Confidence in staff competence	187	31	69
Staff help with motivation	188	29	71
Staff available when needed	186	24	76

Table 3 shows the distribution of responses to items measuring satisfaction with ancillary services. About the half of the sample gave high satisfaction scores to treatment provided for physical and mental health conditions. The proportion of patients who were satisfied with their treatment received in the areas of housing, financial issues, and employment was significantly lower, with only about a quarter reporting “High satisfaction”.

**Table 3.** Percentage of low and high responses on items measuring satisfaction with specific treatment domains.

Items	*n*	% Low satisfaction	% High satisfaction
Physical health treatment	173	35	65
Mental health treatment	175	46	54
Housing support	133	74	26
Financial issues support	143	72	28
Employment support	109	77	23

Patient demographics (age, gender, and polysubstance use) were not significantly associated with patients’ perceived outcome. Ten satisfaction items were significantly correlated with outcome (*p* < 0.01). The item with the highest correlation was confidence in staff competence (*r* = 0.49). Other moderately high correlation coefficients included items measuring opportunities to affect treatment plan (*r* = 0.47) and treatment tailored to individual needs (*r* = 0.45), respectively. Among the five items measuring satisfaction with ancillary services, only satisfaction with help provided for mental health problems was significantly correlated with outcome (*r* = 0.35). The independent variables were only moderately intercorrelated, ranging from *r* = 0.07 to *r* = 0.53.

Table 4 shows the results of multivariate linear regression analysis. As shown in Table 4, the proportion of the variance in the outcome measure explained by the independent variables in the multivariate model was 44%. Two satisfaction items accounted for a significant proportion of the variance in the outcome measure. Patients who reported high satisfaction scores on items measuring confidence in staff competence and opportunities to affect treatment plan were more likely to report a positive outcome of their treatment.

**Table 4.** Results of multivariate linear regression analysis. Domains of treatment satisfaction associated with perceived outcome.

Variables	Multivariate regression, Adj. *R* ^2^ = 0.44		
	Beta	95% CI	*p*-value
Admission process	–0.01	–0.71–0.60	0.870
Information about ward routines	0.11	–0.04–0.77	0.070
Treatment tailored to needs	0.13	–0.07–0.89	0.090
Opportunities to affect treatment plan	0.25	0.42–1.46	< 0.001
Treated with courtesy and respect	0.07	0.28–0.85	0.320
Availability of staff counselling	0.08	–0.18–0.74	0.230
Problems understood by staff	–0.06	–0.71–0.31	0.440
Confidence in staff competence	0.35	0.77–1.78	< 0.001
Staff providing help with motivation	0.08	–0.16–0.72	0.210
Mental health treatment	0.10	–0.11–0.73	0.140

## Discussion

This study explored associations between domains of treatment satisfaction and patient-reported outcome of inpatient treatment for substance abuse. Patients’ confidence in staff competence was the domain of treatment satisfaction most strongly associated with patient-reported outcome. This finding is in accordance with research suggesting that patients’ trust in staff skills and knowledge is important for their progress during substance use treatment (von Greiff & Skogens, 2014). As expected, we also found that satisfaction with opportunities to affect the treatment plan was significantly associated with positive treatment outcomes. The patient’s right to participate and have an active role in the treatment decision-making process is highlighted in the Norwegian Patients’ Right Act (1999, §3-2, §3-2) (Ministry of Health and Care Services, 2016). Our findings are also congruent with studies reporting that patient involvement and participation in drug treatment is important for clinical progress (Brener et al., 2009; McCallum et al., 2015; Morris & Gannon, 2008; Rance & Treloar, 2015). Previous research has also suggested that a qualitatively good relationship between patient and counsellor is a prerequisite for the implementation of user participation in drug treatment (Bryant, Saxton, Madden, Bath, & Robinson, 2008). Thus, it may be suggested that the quality of the patient–counsellor relationship affects patients’ opportunities to participate actively in the treatment process and make their own decisions. Confidence in professional competence may occur when there is a good quality patient–counsellor relationship. The beneficial effect of the therapeutic alliance in the treatment of substance abuse has been documented in previous research (Allen & Olson, 2015; Meier, Barrowclough, & Donmall, 2004; Meier, Donmall, McElduff, Barrowclough, & Heller, 2006; Urbanoski, Kelly, Hoeppner, & Slaymaker, 2012).

Direct comparisons of satisfaction scores between studies are problematic due to the diversity of patient populations and treatment settings and the use of overall satisfaction measures. Nonetheless, our results are consistent with previous research showing that patients in substance abuse treatment are satisfied with staff and programme-related aspects of treatment (Carlson & Gabriel, 2001; Zhang et al., 2008). However, we found that a significant proportion of patients were not satisfied with ancillary services provided, which suggests that their treatment needs were not met in these areas. In particular, patients were dissatisfied with support provided for housing, financial issues, and employment, which are important in the recovery process (Duffy & Baldwin, 2013; Laudet & White, 2010; Nordfjærn et al., 2010). Reduction or cessation of drug use is the primary outcome of substance abuse treatment. However, treatment should also aim to provide help in areas of life that are important for recovery and social reintegration. Thus, preparing the patient for discharge and further follow-up treatment is an important aspect of the treatment approach (Norwegian Directorate of Health, 2015; Sumnall & Brotherhood, 2012). Interagency collaboration between drug treatment services and community-based social services may be essential to ensure that patients have access to housing and social support services upon discharge from inpatient treatment. The present results may reflect disagreements about responsibilities, or otherwise ineffective collaboration between the services.

### Limitations

Our study has some limitations that should be considered. The questionnaire was based on an instrument developed for the measurement of satisfaction among patients being treated for substance abuse in Norway, and has not been tested in previous research. However, the Cronbach’s alpha indicates that the scale has high internal consistency. The outcome measure was based on patient-reported data shortly before discharge from treatment. We do not provide additional objective measures of treatment results, nor data about long-term substance use outcomes. There is therefore some uncertainty about the validity of the present outcome measure. Although previous research has shown that a single item measuring patient-perceived overall helpfulness of treatment at discharge may predict subsequent improvements (Zhang et al., 2008), more research is needed to confirm the associations between specific satisfaction items and treatment outcome found in the present study. Furthermore, patients’ satisfaction with treatment, their assessments of comprehensive services provided and perceived treatment outcome were measured concurrently. Hence, the present data do not imply causality.

Another limitation pertains to the study sample and the moderate response rate. We do not have information about eligible patients who did not participate in the study. Thus, we cannot dismiss the possibility that the sample is biased and that the more dissatisfied patients were not included. Furthermore, the data on the associations between treatment satisfaction and perceived outcome are restricted to treatment completers. The data may therefore be biased, and the results cannot be generalised to all patients entering and receiving residential addiction treatment. Previous research has shown that both patient-related and treatment-related factors are associated with non-completion of substance use treatment (e.g., Curran, Stecker, Han, & Booth, 2009; Palmer, Murphy, Piselli, & Ball, 2009). Inclusion of those who discontinued treatment could have altered the results of this study. However, the design of the study was chosen because we wanted satisfaction measures to reflect the entire inpatient treatment process, including initiatives related to housing, financial issues, and employment, which are typically more in focus towards the end of the patient’s stay.

## Conclusions

The present study demonstrates some detailed associations between treatment satisfaction and self-reported outcomes at treatment completion. Such studies are important because previous research has shown that patient satisfaction is linked to subsequent outcomes (Kendra et al., 2015; Morris & McKeganey, 2007; Perreault et al., 2010). Our findings add to this knowledge that patient-experienced improvements are closely connected to confidence in staff competence and user involvement. Confidence in professional competence is a prerequisite for a therapeutic alliance, which in turn may affect opportunities for meaningful patient participation. However, more research is necessary to explore the underlying causes of the associations found between treatment satisfaction domains and patient-reported outcome.
